# Analysis of risk factors for the increased incidence of gallstone caused by hepatectomy: A retrospective case–control study

**DOI:** 10.3389/fsurg.2023.1097327

**Published:** 2023-03-01

**Authors:** Jiangmin Zhou, Lin Chen, Zhiwei Zhang, Biao Wu

**Affiliations:** ^1^ Department of Hepatobiliary Surgery, Wuhan No.1 Hospital (Wuhan Hospital of Traditional Chinese and Western Medicine), Wuhan, China; ^2^ Hepatic Surgery Center, Tongji Hospital, Tongji Medical College, Huazhong University of Science and Technology, Wuhan, China

**Keywords:** primary liver cancer, benign tumor, hepatectomy, gallstone, retrospective case–control study

## Abstract

**Background:**

An increased risk of gallstones has been observed in patients undergoing hepatectomy. This study attempted to analyze the risk factors for gallstones after hepatectomy.

**Methods:**

From January 2013 to December 2016, clinical data of 1,452 eligible patients who underwent hepatectomy were consecutively reviewed. According to the imaging, including gallbladder ultrasound, computerized tomography, and magnetic resonance imaging, all patients were divided into the gallstone group and the nongallstone group. Univariate and multivariate logistic regression analyses were performed to select indicators associated with gallstone formation among patients after hepatectomy.

**Results:**

In the total sample of included patients, there were 341 patients with gallstones and 1,147 patients without gallstones. The incidence of gallstones was 23.5% (341/1,452). The incidence of gallstones in the primary liver cancer group was higher than that in the benign liver tumor group (25.7% vs. 18.9%, *P* = 0.004). Univariate and multivariate logistic regression analyses showed that female gender, high body mass index, tumor located in S5, and severe postoperative complication were factors related to gallstones in patients with benign liver tumors after hepatectomy. In addition, Child–Pugh B, low albumin, liver cirrhosis, and transcatheter arterial chemoembolization (TACE) after recurrence were factors related to gallstones in patients with primary liver cancer after hepatectomy.

**Conclusions:**

Hepatectomy increased the risk of gallstones in benign or malignant liver tumors, especially when the tumor was located in S5. TACE further increased the risk of gallstones in patients with postoperative recurrence.

## Introduction

The incidence of gallstones in adults is 10%–20% (1–3). The morbidity of gallstones in patients with chronic liver disease (CLD) ranges from 3.6% to 46%, with a 1.2- to 5-fold increase compared with the general population (1, 3–5). Clinically, we have found that the incidence of gallstones is significantly increased in those patients who have undergone liver resection or other therapies such as transcatheter arterial chemoembolization (TACE). Previous studies have shown that any factors involving changes in the composition of hepatic bile and gallbladder hypomotility will facilitate gallstone formation (6–8). The anatomy of the intrahepatic bile duct is complex and closely related to liver tissue. It is inevitable that hepatectomy or TACE treatment will lead to iatrogenic biliary tract injury including the nourishing blood vessels of the biliary tract or the function of the gallbladder's vagus. In addition, whether postoperative complications such as abdominal adhesion or abdominal infection can lead to impaired function of contraction of the gallbladder still needs to be further verified. Therefore, we retrospectively analyzed the risk factors for gallstone formation after hepatectomy.

## Materials and methods

### Patients and definitions

**Figure 1:**
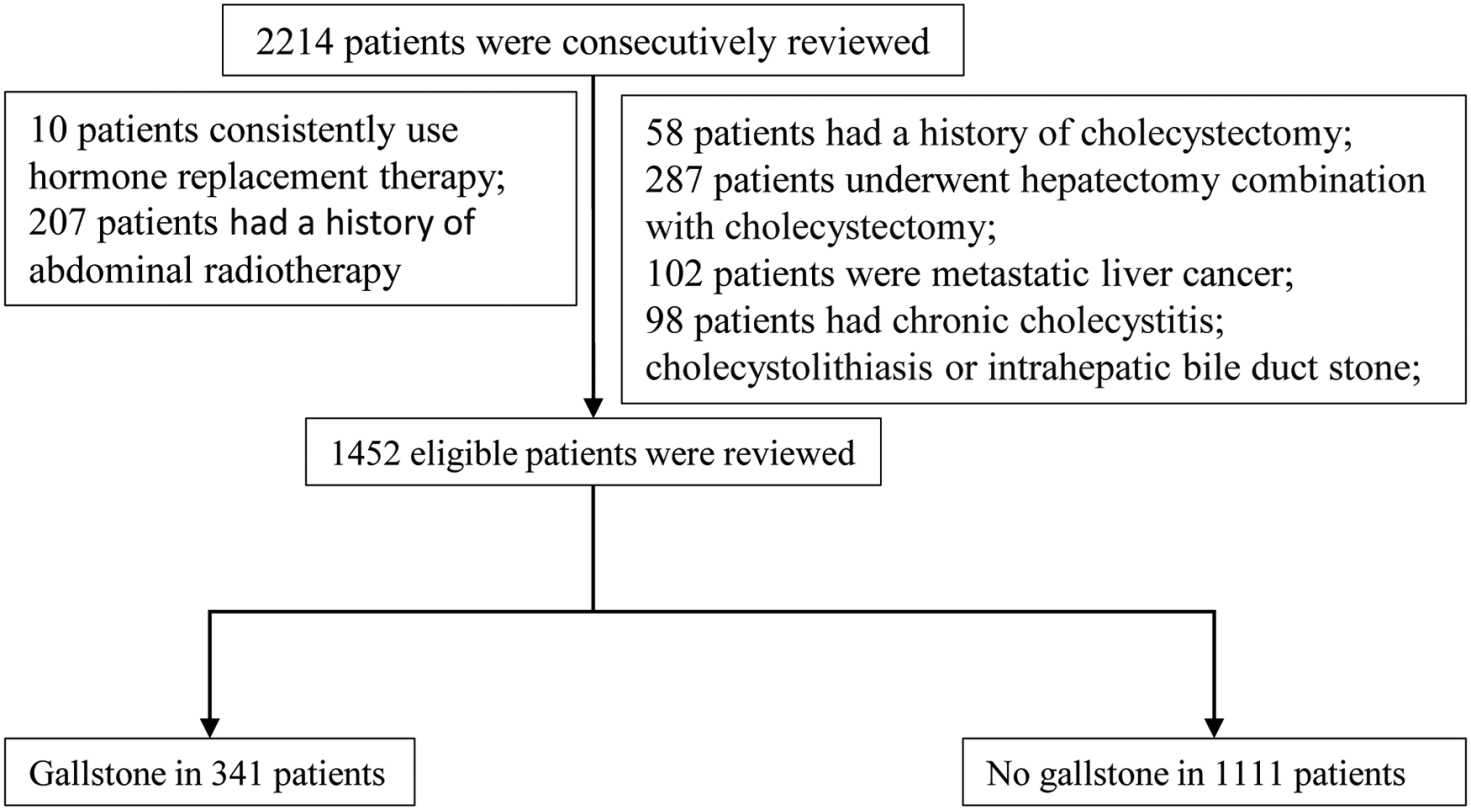
Flow diagram for enrolling patients.

This was a retrospective case–control study. We consecutively reviewed 1,452 eligible patients who underwent hepatectomy between January 2013 and December 2016 in the Hepatic Surgery Center of Tongji Hospital, Tongji Medical College, Huazhong University of Science and Technology. According to the imaging, including gallbladder ultrasound, computerized tomography (CT), and magnetic resonance imaging (MRI), all patients were divided into the gallstone group and the nongallstone group. The flow chart of patients is displayed in Figure 1.

The inclusion criteria are as follows: (1) Child–Pugh A or B; (2) primary liver cancer Barcelona Clinic Liver Cancer (BCLC) A/B stages; and (3) benign tumor of liver including hepatic adenoma, hepatic hemangioma, and hepatic focal nodular hyperplasia (FNH). The exclusion criteria are as follows: (1) metastatic liver cancer; (2) history of cholecystectomy; (3) already existing cholecystitis or gallstones; (4) intrahepatic bile duct stones; (5) consistent use of hormone replacement therapy; and (6) abdominal radiotherapy.

The gallbladder is attached to S5 of the liver. In general, anatomical hepatectomy usually combines with cholecystectomy when a tumor is located in S5 of the liver. When the tumor is small and nonanatomic hepatectomy is performed, gallbladder preservation is feasible. Thus, there is a considerable number of patients with tumors located in S5 of the liver in our study who underwent hepatectomy with gallbladder preservation.

Postoperative complications are common after hepatectomy. The severity of complications was classified according to the Clavien–Dindo classification system (9). Grade I complications included mild pleural, peritoneal effusion, electrolyte disturbance, and other deviations from the normal postoperative course. Grade II complications included blood transfusion, plasma infusion, or albumin infusion. Grade III covered severe pleural and peritoneal effusion requiring percutaneous aspiration or drainage. Grade IV complications covered severe lung infections, respiratory failure, or kidney failure, requiring intubation or dialysis treatment.

### Surgical methods

Hepatectomy was accomplished in a hepatic surgery center, Tongji Hospital, Tongji Medical College, Huazhong University of Science and Technology. All patients were given general anesthesia. The methods of hepatectomy included open and laparoscopic hepatectomy. Partial resection or anatomic resection of the liver was performed based on the tumor location. Complete removal of at least one Couinaud segment containing the focus and the portal vein in the drainage area of the lesion was defined as an anatomic resection. A complete tumor plus a rim of non-neoplastic liver parenchyma was considered a nonanatomic resection. Partial resection makes preservation of the gallbladder possible when the surgeon removes the small lesions located at S5.

The study was approved by the Ethical Committee of Tongji Hospital of Tongji Medical College of Huazhong University of Science and Technology. All procedures performed in this study abided by the Declaration of Helsinki.

### Statistical analysis

Continuous variables were presented as the median and interquartile range (IQR), and categorical data as were presented numbers and percentages. A *χ*^2^ test and the Mann–Whitney *U* test were used to compare groups where appropriate. Univariate and multivariate logistic regression analyses were performed to evaluate risk factors for gallstone development. *P < *0.05 was considered statistically significant. Statistical analyses were performed using SPSS version 19.0 for Windows (SPSS, Chicago, IL, United States).

## Results

### Clinical characteristics of patients

**Figure 2:**
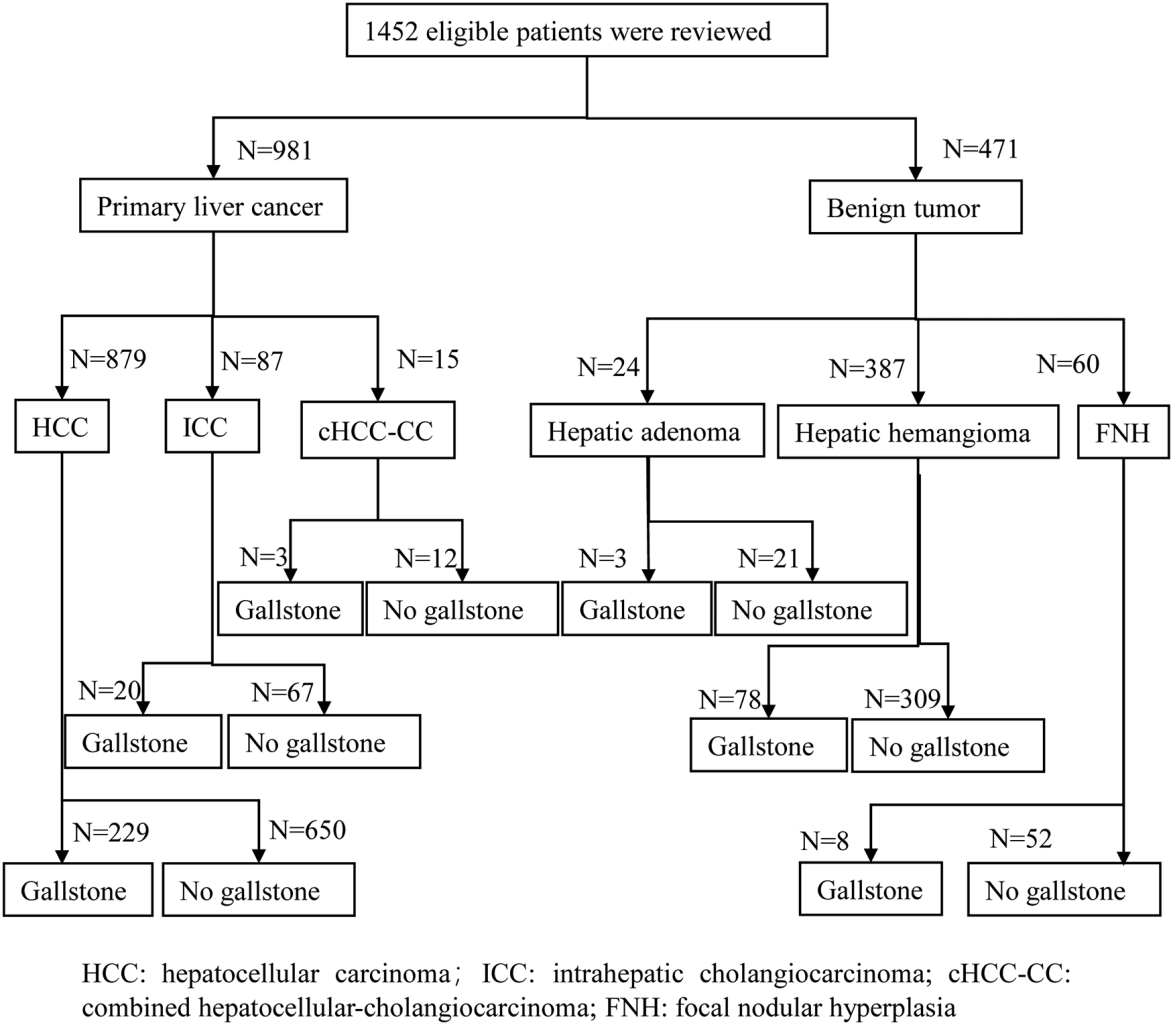
Proportion of gallstones in malignant and benign tumor groups.

Figure 2 shows that 1,452 eligible patients were stratified according to the malignant or benign tumor. In the total sample of included patients, there were 981 patients with primary liver cancer and 471 patients with benign tumors, including 24 patients with hepatic adenoma, 387 patients with hepatic hemangioma, and 60 patients with FNH.

The median age was 56 years, and 85.5% (1,241/1,452) of patients were men. In total, 96.6% (1,403/1,452) of patients were Child–Pugh A. The etiology of patients was 776 (53.4%) positive for hepatitis B virus (HBV), 18 (1.2%) positive for hepatitis C virus (HCV), 41 (2.8%) for other causes, and 617 (42.6%) with no hepatitis background. A higher body mass index (BMI), total bilirubin, tumor size, and lower albumin were observed in the gallstone group. The proportion of the female gender, diabetes, Child–Pugh B, hepatitis B surface antigen (HBsAg)-positive, liver cirrhosis, S5 liver resection, multiple tumor, and postoperative complications was higher in the gallstone group (Table 1).

**Table 1:** Baseline characteristics of patients.

Clinical characteristics	Gallstone (*n* = 341)	No gallstone (*n* = 1,111)	*P*
Age (years), M (IQR)	57 (49–65)	56 (47–67)	0.252
Sex, female, *N* (%)	85 (25.1)	126 (11.3)	<0.001
BMI (kg/m^2^), M (IQR)	25.1 (23.4–28.6)	22.5 (21.1–23.5)	0.015
Diabetes, yes, *N* (%)	45 (15.8)	67 (6.0)	<0.001
Smoking, yes, *N* (%)	152 (44.6)	456 (41.0)	0.248
Drinking, yes, *N* (%)	120 (35.2)	378 (34.0)	0.691
Child–Pugh, B, *N* (%)	32 (9.4)	17 (1.5)	<0.001
HBsAg, positive, *N* (%)	310 (90.9)	466 (41.9)	<0.001
ALT (U/L), M (IQR)	34 (19–53)	32 (22–53)	0.247
AST (U/L), M (IQR)	31 (16–49)	29 (18–54)	0.145
Albumin (g/L), M (IQR)	35.1 (33.8–38.4)	37.1 (35.1–40.2)	0.021
TBiL (μmol/L), M (IQR)	14.2 (10.6–19.6)	10.2 (7.2–16.2)	<0.001
Liver cirrhosis, yes, *N* (%)	315 (92.5)	445 (40.1)	<0.001
Resection types, AR, *N* (%)	98 (28.7)	333 (30.0)	0.663
Surgical methods, open *N* (%)	140 (41.1)	467 (42.0)	0.749
Liver resection, S5, *N* (%)	119 (34.9)	88 (7.9)	<0.001
Tumor size (cm), M (IQR)	5.6 (3.8–6.4)	4.2 (3.0–5.5)	0.017
Tumor number, multiple, *N* (%)	106 (31.1)	100 (9.0)	<0.001
Complications, yes, *N* (%)	219 (64.2)	267 (24.0)	<0.001

M, median; IQR, interquartile range; BMI, body mass index; HBsAg, hepatitis B surface antigen; ALT, alanine aminotransferase; AST, aspartate aminotransferase; TBiL, total bilirubin; AR, anatomic resection.

The overall gallstone incidence was 23.5% (341/1,452), and the asymptomatic gallstone incidence was 83.6% (285/341). The incidence of gallstones in the primary liver cancer group was 25.7% (252/981) higher than that in the benign tumor group [18.9% (89/471), *P* = 0.004]. Asymptomatic gallstones accounted for 81.7% (206/252) of all gallstones in the primary liver cancer group and 89.2% (79/89) of all gallstones in the benign tumor group (*P* = 0.11).

In 4 years, 41 out of 341 patients (12.0%) with gallstones underwent cholecystectomy. All patients with cholecystectomy had significant right upper abdominal pain, and their Murphy sign was positive. In addition, CT indicated thickening of the gallbladder wall and chronic cholecystitis. No cholecystectomy was performed for all asymptomatic and some symptomatic gallstones. Moreover, we had not observed any serious disease caused by gallstones, including acute pancreatitis and gallbladder cancer.

### Factors associated with a gallstone in patients with benign liver tumors

Univariate and multivariate logistic regression analyses showed that female gender (OR: 1.547, 95% CI: 1.358–2.356, *P* = 0.018), BMI (OR: 3.255, 95% CI: 2.789–4.589, *P* = 0.009), liver resection at S5 (OR: 3.687, 95% CI: 2.987–4.698, *P* = 0.012), and postoperative complication (OR: 3.684, 95% CI: 3.489–4.398, *P* < 0.001) were factors related to gallstone in patients with benign liver tumors after hepatectomy (Table 2).

**Table 2:** Univariate and multivariate *logistic regression* analyses on factors associated with gallstones for patients with benign liver tumors.

Variables	Univariate analysis		Multivariate analysis	
	OR (95% CI)	*P*	OR (95% CI)	*P*
Age (years), M (IQR)	0.998 (0.997–1.002)	0.854		
Female gender, *N* (%)	1.297 (1.181–1.727)	0.021	1.547 (1.358–2.356)	0.018
BMI (kg/m^2^), M (IQR)	4.512 (3.212–6.589)	<0.001	3.255 (2.789–4.589)	0.009
Diabetes, *N* (%)	1.214 (1.123–1.689)	0.019	1.123 (0.998–1.136)	0.148
Smoking, *N* (%)	1.112 (0.987–1.245)	0.265		
Drinking, *N* (%)	1.089 (0.975–1.102)	0.239		
ALT (U/L), M (IQR)	0.989 (0.971–1.036)	0.687		
AST (U/L), M (IQR)	0.997 (0.974–1.021)	0.876		
Albumin (g/L), M (IQR)	1.258 (1.025–1.359)	0.025	1.025 (0.996–1.285)	0.365
TBiL (μmol/L), M (IQR)	1.021 (0.991–1.121)	0.129		
Resection types, AR, *N* (%)	1.009 (0.986–1.158)	0.259		
Surgical methods, Open *N* (%)	1.008 (0.965–1.032)	0.298		
Liver resection, S5, *N* (%)	2.358 (1.532–3.021)	0.027	3.687 (2.987–4.698)	0.012
Tumor size (cm), M (IQR)	1.002 (0.986–1.063)	0.521		
Tumor number, Multiple, *N* (%)	1.021 (0.983–1.125)	0.121		
Complications, *N* (%)	4.215 (2.879–6.328)	<0.001	3.684 (3.489–4.398)	<0.001

M, median; IQR, interquartile range; BMI, body mass index; ALT, alanine transaminase; AST, aspartate aminotransferase; TBiL, total bilirubin; AR, anatomic resection.

### Factors associated with gallstones in patients with primary liver cancer

Univariate and multivariate logistic regression analyses showed that female gender (OR: 2.314, 95% CI: 2.021–4.529, *P* = 0.037), BMI (OR: 2.125, 95% CI: 1.546–3.356, *P* = 0.011), diabetes (OR: 1.846, 95% CI: 1.259–2.136, *P* = 0.019), Child–Pugh B (OR: 2.472, 95% CI: 1.261–4.025, *P* = 0.008), albumin (OR: 2.851, 95% CI: 2.622–3.914, *P* < 0.001), liver cirrhosis (OR: 4.258, 95% CI: 3.258–6.398, *P* < 0.001), liver resection at S5 (OR: 4.589, 95% CI: 3.514–6.215, *P* < 0.001), postoperative complication (OR: 6.256, 95% CI: 5.021–7.654, *P* < 0.001), and TACE after recurrence (OR: 2.691, 95% CI: 2.102–3.028, *P* = 0.019) were factors related to gallstones in patients with primary liver cancer after hepatectomy (Table 3).

**Table 3:** Univariate and multivariate *logistic regression* analyses on factors associated with gallstones for patients with primary liver cancer.

Variables	Univariate analysis		Multivariate analysis	
	OR (95% CI)	*P*	OR (95% CI)	*P*
Age (years), M (IQR)	0.988 (0.977–1.008)	0.258		
Female gender, *N* (%)	1.587 (1.459–2.585)	0.029	1.314 (1.021–1.929)	0.037
BMI (kg/m^2^), M (IQR)	2.452 (1.896–3.598)	0.009	2.125 (1.546–3.356)	0.011
HBsAg, *N* (%)	1.895 (1.199–2.205)	0.028	1.105 (0.831–1.469)	0.125
Diabetes, *N* (%)	1.657 (1.451–1.982)	0.024	1.846 (1.259–2.136)	0.019
Smoking, *N* (%)	1.012 (0.993–1.142)	0.241		
Drinking, *N* (%)	1.029 (0.974–1.203)	0.313		
Child–Pugh B, *N* (%)	2.595 (1.377–4.889)	0.003	2.472 (1.261–4.025)	0.008
ALT (U/L), M (IQR)	0.996 (0.994–1.025)	0.837		
AST (U/L), M (IQR)	0.998 (0.991–1.018)	0.785		
Albumin (g/L), M (IQR)	3.657 (2.547–6.358)	<0.001	2.851 (2.622–3.914)	<0.001
TBiL (μmol/L), M (IQR)	1.254 (1.125–2.541)	0.029	1.125 (0.996–1.358)	0.125
BCLC B stage	2.241 (1.987–2.419)	0.031	1.147 (0.987–1.225)	0.134
Liver cirrhosis, *N* (%)	5.489 (3.532–8.102)	<0.001	4.258 (3.258–6.398)	<0.001
Resection types, AR, *N* (%)	1.084 (0.992–1.095)	0.127		
Surgical methods, Open *N* (%)	1.011 (0.998–1.048)	0.126		
Liver resection, S5, *N* (%)	3.584 (2.698–4.112)	<0.001	4.589 (3.514–6.215)	<0.001
Tumor size (cm), M (IQR)	1.241 (1.023–1.598)	0.029	1.051 (0.874–1.918)	0.254
Tumor number, Multiple, *N* (%)	1.857 (1.259–2.547)	0.021	1.012 (0.798–2.024)	0.122
Complications, *N* (%)	5.236 (3.034–10.752)	<0.001	6.256 (5.021–7.654)	<0.001
Recurrence, *N* (%)	1.354 (1.015–3.987)	0.031	1.248 (0.751–2.145)	0.239
TACE after recurrence, *N* (%)	3.521 (2.398–5.269)	0.002	2.691 (2.102–3.028)	0.019
MWA after recurrence, *N* (%)	1.254 (1.129–2.014)	0.036	1.025 (0.869–2.069)	0.297

M, median; IQR, interquartile range; BMI, body mass index; HBsAg, hepatitis B surface antigen; ALT, alanine transaminase; AST, aspartate aminotransferase; TBiL, total bilirubin; BCLC, Barcelona Clinic Liver Cancer; AR, anatomic resection; TACE, transcatheter arterial chemoembolization; MWA, microwave ablation.

### Typical cases of gallstone stone formation

**Figure 3:**
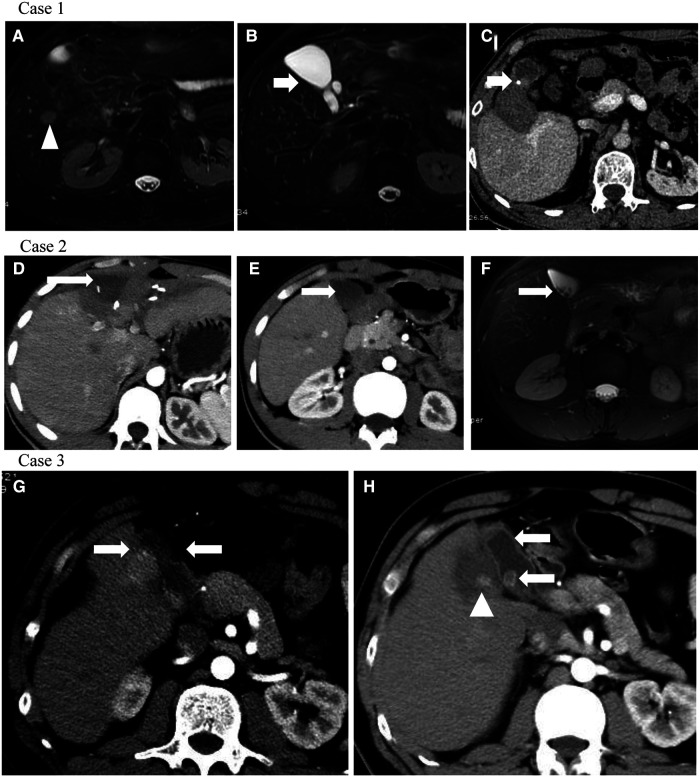
Three typical cases of gallstone formation after hepatectomy or recurrent patients receiving TACE. (**A**) Tumor was located in S5 (white triangle); (**B**) there was no gallstone in the gallbladder before surgery; (**C**) gallstone developed in the gallbladder (arrow), and the gallbladder tightly adhered to the area of abdominal adhesions; (**D,E**) surgical area was located in S4 and partial S5 and the surgical area presented with effusion and adhesions (arrow); (**F**) gallstone developed in the gallbladder ultimately (arrow); (**G**) recurrent patient after surgery whose lesion was located in S5 received TACE treatment; most areas of the lesion are necrotic (white triangle); (**H**) gallstone developed in the gallbladder ultimately and the wall of the gallbladder was significantly thickened (arrow).

Figure 3 shows three typical cases of gallstone formation after hepatectomy or recurrent patients receiving TACE. Case 1 displayed that the tumor was located in S5 (white triangle) and there was no gallstone in the gallbladder before surgery (Figures 3A,B). The gallstone developed in the gallbladder (arrow), and the gallbladder tightly adhered to the area of abdominal adhesions (Figure 3C). Case 2 showed that the surgical area was located in S4 and partial S5, and the surgical area presented with effusion and adhesions (arrow) (Figures 3D,E). The gallstone developed in the gallbladder ultimately (arrow) (Figure 3F). Case 3 showed that a recurrent patient after surgery whose lesion was located in S5 received TACE treatment; most areas of the lesion are necrotic (white triangle). The gallstone developed in the gallbladder ultimately and the wall of the gallbladder was significantly thickened (arrow) (Figures 3G,H).

## Discussion

The traditional risk factors for gallstone disease are the four “F's: female, fat, forty, and fertile,” with many studies supporting the known risk factors for gallstone disease (10, 11). In our study, female gender and BMI are risks factor for the formation of gallstones in patients with benign or malignant tumors after hepatectomy. BMI, which positively correlates with blood lipids, is also a well-known risk factor for gallstones (12, 13). A cohort has confirmed a close association (in both genders, but especially pronounced in women) between increasing BMI and increased risk of symptomatic gallstone disease (14). In addition, it was reported that insulin resistance is closely related to gallstone formation (15–17). In the study, diabetes was observed to be associated with gallstone formation in malignant tumor patients but not in patients with benign tumors.

Interestingly, in our study, the incidence of gallstones after hepatectomy was significantly higher in patients with primary liver cancer than that in patients with benign liver tumors. Generally speaking, most patients with primary liver cancer are accompanied by chronic liver disease including cirrhosis or hypoalbuminemia. It has been reported that liver cirrhosis has been identified as another risk factor for the formation of gallstones, and more severe cirrhosis and longer cirrhosis duration result in a higher incidence of gallstones (18–20). Previous studies have shown that a thickened gallbladder wall and impaired contractility have been observed when patients present with liver cirrhosis, hepatic failure, and portal hypertension (21–24). It has been reported that hypoalbuminemia will increase tissue edema, including the gallstone, which leads to impaired gallstone motility and contractility and provides a potential pathophysiologic basis for gallstone formation (24). In addition, patients with more severe chronic liver disease are more likely to have postoperative complications. Severe surgical complication grade increased the duration of parenteral nutrition and prolonged fasting, resulting in bile concentration in the gallstone and impaired gallstone motility. Peritoneal effusion, peritoneal cavity infection, and bile leakage often indicated the existence of serious complications contributing to a severer inflammatory response in the abdominal cavity and severer abdominal organ adhesions, leading to swelling and exudation of local tissues and impairing gallstone motility.

In addition, we observed an increased risk of gallstone formation when the tumor is located in S5. Gallstone function was extremely affected when the behavior of liver resection was related to the gallbladder. On the one hand, peritoneal effusion or abdominal adhesion will inevitably occur near the gallbladder, which will impair its motility. On the other hand, when hepatectomy is performed near the gallbladder, it may be disturbed (for instance, it may be torn or squeezed), resulting in gallbladder motility and contractility.

In our study, we found that patients receiving TACE for recurrence after hepatectomy had a higher incidence of gallstones. On the one hand, as we know, the gallbladder artery arises from the right hepatic artery. When TACE is performed, the embolization agent or chemotherapy drug may reflux into the gallbladder artery, leading to a decreased blood supply to the gallbladder and mucosal necrosis of the gallstone; this is more likely when TACE is performed *via* the right hepatic artery. Therefore, decreased blood supply to the gallbladder leads to gallstone hypomotility and eventually to gallbladder atrophy. On the other hand, chemotherapy drugs stimulate the gallbladder mucosa for a long period, such that the gallbladder mucosa thickens and chronic cholecystitis develops.

## Conclusion

In conclusion, the retrospective case–control study suggests an association between the incidence of gallstones and the location of tumors, especially in S5. In addition, for patients with hepatocellular carcinoma (HCC), TACE further increases the risk of gallstones in patients with postoperative recurrence. Thus, the increased risk of gallstone development after hepatectomy deserves to be concerned.

## Data Availability

The datasets presented in this study can be found in online repositories. The names of the repository/repositories and accession number(s) can be found here: 10.6084/m9.figshare.21505629.
